# Exploring differential production of alkaloids and terpenoids in *Cristataspora coffeata* (Berk:) Robledo (Ganodermataceae) under submerged culture

**DOI:** 10.1371/journal.pone.0337315

**Published:** 2025-12-31

**Authors:** Ricardo A. González-Hernández, Rodrigo Villanueva-Silva, C. Andrés Arganis-Ramírez, Norma A. Valdez-Cruz, Mauricio A. Trujillo-Roldán

**Affiliations:** 1 Posgrado en Ciencias Biológicas, Universidad Nacional Autónoma de México, México. Unidad de Posgrado, Edificio B, 1° Piso, Circuito de Posgrados, Ciudad Universitaria, Coyoacán, C.P, Mexico City, México; 2 Centro de Nanociencias y Nanotecnología, Universidad Nacional Autónoma de México, Km 107 carretera Tijuana-Ensenada, Baja California, México; 3 Departamento de Biología Molecular y Biotecnología, Instituto de Investigaciones Biomédicas, Universidad Nacional Autónoma de México, Ciudad Universitaria, Coyoacán, C.P, Ciudad de México, México; 4 Departamento de Toxicología, Centro de Investigación y Estudios Avanzados del Instituto Politécnico Nacional. Unidad Zacatenco, Av. Instituto Politécnico Nacional, San Pedro Zacatenco, Gustavo A. Madero, C.P, Ciudad de México, México; 5 Programa de Investigadoras e Investigadores por México. Secretaría de Ciencia, Humanidades, Tecnología e Innovación. Av. Insurgentes Sur, Crédito Constructor, Benito Juárez, C.P, Ciudad de México, México; 6 Facultad de Química, Universidad Nacional Autónoma de México, Ciudad Universitaria, Coyoacán, C.P, Ciudad de México, México; Benemérita Universidad Autónoma de Puebla: Benemerita Universidad Autonoma de Puebla, MEXICO

## Abstract

Basidiomycetes are recognized for their capacity to produce a diverse range of secondary metabolites, particularly terpenoids and alkaloids, which have significant applications in the pharmaceutical, agricultural, and chemical industries. Among them, *Cristataspora* (formerly *Humphreya*) *coffeata* has attracted attention for its promising biosynthetic potential. Investigating the range of compounds produced by this fungus in controlled submerged culture conditions, rather than in the fruiting body stage, is particularly beneficial, as it enables precise control of environmental and nutritional factors, resulting in more consistent and scalable metabolite production. This study examined the differences in the cultivation of *C. coffeata* and the synthesis of terpenoids and alkaloids when the growth medium was supplemented with either glucose or lactose as a carbon source. It was observed that when the fungus was cultivated with glucose, the kinetic parameters did not differ from those with lactose. However, the individual pellet size was larger with glucose compared to lactose. Regarding secondary metabolites, in glucose cultures, a differential production of terpenoids and alkaloids was observed in the biomass and culture supernatant at 6 and 14 days, respectively, compared to lactose, as determined by TLC and spectrophotometric quantification. GC-MS analysis revealed that 19 differential compounds were detected in the biomass and 9 in the supernatant when glucose was used as the carbon source. In contrast, with lactose, 14 compounds were differentially produced in the biomass and 7 in the supernatant. These findings underscore the relevance of submerged cultivation for modulating secondary metabolite profiles and the importance of selecting the appropriate carbon source to maximize yields of target compounds.

## Introduction

Basidiomycetes are highly relevant organisms due to their potential applications in various industrial sectors, such as pharmaceuticals and food, resulting from the secondary metabolites they produce. Among the primary bioactive metabolites produced by these fungi are polysaccharides, terpenoid lipids, and alkaloids. For example, in the case of terpenoids, their applications extend to synthesis precursors, flavorings, fuels, and multiple pharmacological activities [1–4]. Likewise, fungal alkaloids exhibit pharmacological activities of relevance, including antidepressant, antineurodegenerative, cytotoxic, and anti-inflammatory properties [5–7]. For this reason, greater understanding of the production, extraction, and characterization of these compounds in novel species are significant for pharmaceutical, food, fuel, and other industries [7–10]. In fungi, submerged cultivation offers substantial advantages over harvesting metabolites from fruiting bodies, as it allows precise control of culture conditions, improves reproducibility, and enhances scalability, while also enabling the exploration of metabolic diversity under laboratory conditions [11–14]. An approach implemented for the exhaustive search for molecules produced by this type of organism is the OSMAC (One Strain Many Compounds) strategy, which is based on the use of different culture conditions that include agitation, aeration, pH, salinity, and nutrients, among others [15,16]. In the case of fungi, it has been observed that the use of different carbon sources impacts the production of terpenoids and alkaloids; this is because these parameters influence the regulation of some synthases in metabolic pathways [17,18].

Conversely, the fungus *Cristataspora* (formerly *Humphreya*) *coffeata* is a basidiomycete closely associated with the Ganodermataceae family, renowned for its extensive production of fungal secondary metabolites. However, despite this phylogenetic relationship, the biosynthetic capabilities of *C. coffeata* remain largely unexamined, particularly concerning the synthesis of alkaloids and terpenoids, for which no previous studies have been documented. In an in vitro study carried out in our group, it was demonstrated that supernatant extracts from submerged cultures of *C. coffeata* have cytotoxic activity on the Jurkat lymphoma cell line (from 250–2500 µg/mL) without affecting the HaCaT keratinocyte line, or cervical cancer lines (HeLa and InBl) [19]. Moreover, the study demonstrated that culture conditions (particularly the type and concentration of carbon and nitrogen sources, as well as the initial pH) have a significant impact on fungal growth and exopolysaccharide (EPS) production. This raises an interesting question as to whether the nature of the carbon source used also influences the differential production of alkaloids and terpenoids.

## Materials and methods

### Submerged culture of *C. coffeata*

*C. coffeata* was obtained from the culture collection of the Universidad de Antioquia (Medellín, Colombia), originally isolated from fruiting bodies collected in Tierra Alta, Córdoba (northern Colombia). The strain was subsequently co-cultured and preserved at the Instituto de Investigaciones Biomédicas, Universidad Nacional Autónoma de México (UNAM, in Mexico City, Mexico), and at the Centro de Nanociencias y Nanotecnología (UNAM, in Ensenada, B.C., Mexico). For long-term conservation, mycelial fragments were stored in sterile distilled water at room temperature, following the published method for *C. coffeata* [20].

*C. coffeata* was reactivated on solid medium containing (in g/L): yeast extract (5), peptone (5), KH_2_PO_4_•H_2_O (1.0), MgSO_4_•7H_2_O (0.5), agar (20), and glucose (50). For cultures in liquid medium, the one reported by Porras-Arboleda et al. [19] was used as a base, which contains (in g/L): yeast extract (5), peptone (5), KH_2_PO_4_•H_2_O (1.0), MgSO_4_•7H_2_O (0.5), Vitamin B1 (0.05) and the carbon source (glucose or lactose) in a concentration equivalent to 150 mmol/L of total carbon, all reagents used were from JT Baker (Phillipsburg, NJ, USA) and Sigma-Aldrich (St. Louis, MO, USA).

Submerged cultures were carried out, at least in triplicate, in 250 mL Erlenmeyer shake flasks (Duran, DURAN Group GmbH, Mainz, Germany) with a filling volume of 50 mL, shaken at 150 rpm and 30 °C in a New Brunswick Classic Series C25 incubator (2.54 cm shaking diameter). The cultures for kinetic characterization were maintained for 16 days. Specific cultures were used to obtain biological material, with durations of 6 and 14 days of culture. The average pellet diameter was determined by measuring at least 20 randomly selected pellets with a vernier caliper (±0.1 mm) for each condition [21,22].

### Extracts of biomass and culture supernatant of *C. coffeata*

All solvents used in the extraction process were of HPLC grade, sourced from Chromasolv, Merck, and Sigma-Aldrich (Merck KGaA, Darmstadt, Germany). For extracting intracellular compounds, the biomass was collected by filtration and then washed with deionized water, using three 20 mL volumes, followed by vacuum filtration. It was then left to dry in an unheated oven at a 20 psig vacuum until it reached a constant weight. The solvents used for the extraction were chloroform and methanol, in increasing order of polarity, in four stages. Initially, it began with a 72-h maceration using chloroform, at a ratio of 20 mL per gram of biomass, to ensure the recovery of thermolabile molecules with intermediate-low polarity. The second stage consisted of performing a Soxhlet extraction using chloroform in the same proportion of solvent. In the third stage, maceration was carried out for 72 hours using methanol, followed by a subsequent fourth stage where a Soxhlet extraction was performed using a proportion of 20 mL/g of biomass in both cases.

For extracting compounds from the culture supernatant, a liquid-liquid extraction was carried out, using one-third of the chloroform volume relative to the supernatant volume. Four consecutive extractions were performed, and the resulting extracts were pooled. All extracts were concentrated using a rotary evaporator (Flash Evaporator, Buchler Instruments, Lenexa, KS, USA). The concentrated extract was resuspended in a chloroform:methanol (1:1) solvent mixture and transferred to pre-weighed vials. The extracts were stored at room temperature until chromatographic analysis.

### Kinetic parameters

The specific growth rate (μ) was calculated during the exponential phase by plotting the natural logarithm of biomass concentration [Ln(X)] versus time and performing a linear regression in the linear exponential growth. The slope of the resulting line represents μ. For glucose-supplemented cultures, days 2–5 were analyzed, while for lactose-supplemented cultures, days 3–5 were used.

The maximum biomass produced in the culture (X_max_) is estimated by the difference between the maximum biomass obtained in the culture and the initial biomass at the inoculum. Since the maximum biomass produced value is the one used to calculate the other global kinetic parameters, we decided to display it directly in the kinetic parameters table. The value of the global substrate consumption rate is obtained by subtracting the residual value of the carbon source (glucose or lactose in our case) at the final time from the initial substrate value.

The overall biomass yield on substrate (Y_x/s_) was calculated as the ratio of biomass produced to substrate consumed at the end of the culture. The overall specific substrate consumption rate (q_s_) represents the substrate utilization per unit of biomass per unit time and was determined by dividing the substrate consumption by the maximum biomass achieved, normalized over the culture duration (16 days in this study).

### Determination of alkaloids and terpenoids

The quantification of alkaloids was performed using the Ehrlich method, modified to a 1-mL reaction volume [23]. A standard curve was created using L-tryptophan (Sigma-Aldrich, HPLC grade > 98% purity) in increasing concentrations from 5 μg/mL to 150 μg/mL, prepared from a solution of 1 mg/mL L-tryptophan dissolved in 10 M HCl. The corresponding volume was taken for 5, 10, 20, 40, 60, 80, 100, and 150 μg. 120 μL of the freshly prepared Ehrlich-Van Urk reagent was immediately added, and the volume was adjusted to 1.0 mL with distilled water. Subsequently, they were heated at 80 °C for 60 min and read immediately in a spectrophotometer (λ = 580 nm).

For all samples, 2 mg of each extract was used, and 120 μL of 10 M HCl was added for acid extraction for 10 minutes. Subsequently, the mixture was centrifuged at 11,800 × *g* for 10 min. The supernatant was recovered, and 120 μL of the Ehrlich-Van Urk reagent was added. The mixture was then thoroughly mixed, adjusted to 1.0 mL with distilled water, and heated at 80 °C for 60 minutes.

Regarding the quantification of terpenoids, a reaction with anisaldehyde was employed, based on an adaptation of the method used by Oludemi et al. [24]. The standard curve was prepared by dissolving 25 mg of ursolic acid in absolute ethanol to a final volume of 25 mL, yielding a stock solution of 1000 μg/mL. From this solution, the corresponding volumes of 200, 150, 100, 50, 30, 20, 10, and 5 μg were transferred to 1.5 mL Eppendorf tubes, and 125 μL of anisaldehyde_(AcOH)_ (50% v/v) and 165 μL of concentrated de H_2_SO_4_ were added. The mixture was kept in a water bath (60 °C) for 30 min and immediately adjusted to 1.0 mL with glacial acetic acid, to determine the absorbance at λ = 548 nm.

For each sample, 0.4 mg of extract was taken (in triplicate) and 125 μL of anisaldehyde_(AcOH)_ (50% v/v) and 165 μL of concentrated H_2_SO_4_ were added, following the treatment specified above.

### Comparison of metabolite profiles

A comparison of the compounds produced at different cultivation times in both carbon sources was performed using thin-layer chromatography (TLC) on 10.0 cm x 5.0 cm aluminum plates with Silica 60, F_254_ (Supelco, Merck). For sample preparation, 0.5 mg of each extract was transferred to clean and dry glass vials and subsequently dissolved in a chloroform-methanol mixture (1:1) at a final concentration of 500 µg/mL. The elution systems used were chloroform–methanol (95:5) and methanol–chloroform (7:3). The developing agent used was 10% H_2_SO_4(aq)_. Retention factors (R_f_) were calculated for the major compounds, and the mean and standard deviation for each component were calculated. Ursolic acid was used as the reference terpene.

### Fingerprint of biomass extracts

The fingerprinting of chloroform extracts obtained from *C. coffeata* biomass, grown for 14 days with glucose or lactose as the carbon source, was performed using Atmospheric Pressure Chemical Ionization-Mass Spectrometry (APCI-MS). This technique has proven very useful in the rapid and routine analysis of low-molecular-weight molecules, as well as in complex mixtures, as in our case (Gates, 2021). In addition, the hydrogen nuclear magnetic resonance spectrum (^1^H-NMR), was obtained using deuterated DMSO as solvent and control.

### Analysis by Gas Chromatography–Mass Spectrometry (GC-MS)

To determine the most abundant and differential compounds present in each of the extracts obtained from the *C. coffeata* cultures, an analysis was performed using gas chromatography coupled with mass spectrometry (GC-MS). The equipment used was an Agilent GC 6890, MSD 5973N. The operating conditions consisted of an HP-5 column (30 m x 0.25 mm x 0.25 μm), and Helium was used as the carrier gas with a flow rate of 1 mL/min. The initial oven temperature was 50 °C (1.0 min) and a final temperature of 300 °C (10 min), with a heating rate of 10 °C/min. Electron impact ionization was performed using a quadrupole analyzer (70 eV). The spectra obtained were processed using MSD ChemStation software, with a focus on the most abundant compounds and those that differed between the evaluated conditions.

To compare the composition of the different extracts, an analysis was performed of the number of compounds detected in each extract (total extract content). The number of compounds corresponding to alkaloids or terpenoids in the extract was then counted. Using this information, the percentage abundance of each type of compound in relation to the extract was calculated, which was referred to in this study as relative abundance. Compounds that did not correspond to alkaloids or terpenoids were grouped as others.

### Statistical analysis

Comparisons between samples were first analyzed using one-way ANOVA and Tukey’s test. A Student’s t-test was also used to compare pellet sizes. In both cases, the analyses were considered to have a p-value < 0.05.

## Results

### Characterization of cultures of *C. coffeata*

**Fig 1:**
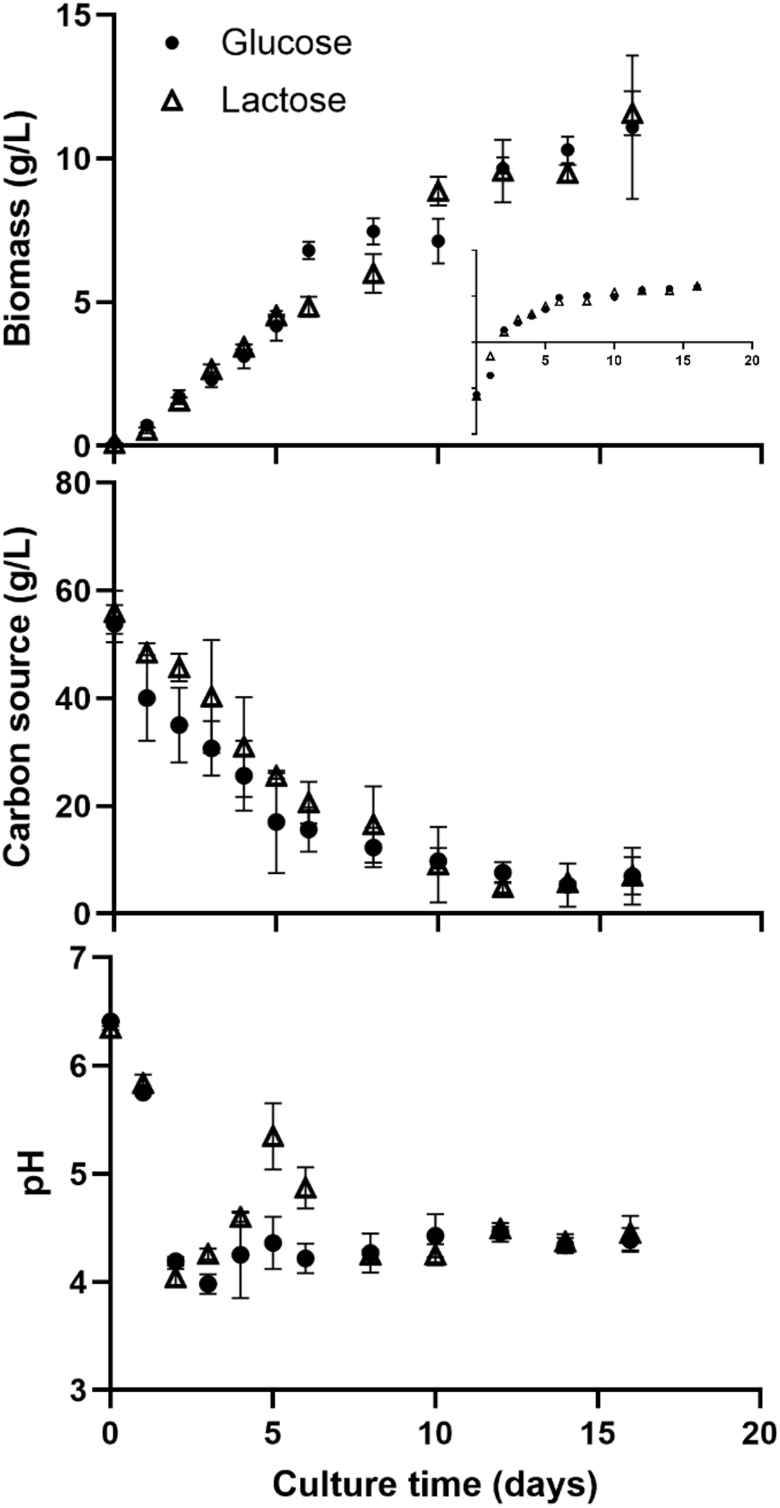
Kinetic characterization of *C. coffeata* growth with each carbon source. Biomass **(a)**, Substrate consumption **(b)**, Medium pH **(c)**. Conditions: 30 °C, 150 rpm, 16 days. No significant differences were found at p < 0.05. n = 3.

The growth parameters evaluated during the cultivation of the fungus with glucose or lactose as a carbon source included maximum biomass (Xm), specific growth rate (μ), specific consumption rates (qs), and pH time-lapse (Fig 1, Table 1). There is no significant difference in μ when using glucose (μ_glu_ = 0.30 ± 0.00 d^-1^) or lactose (μ_lac_ = 0.27 ± 0.03 d^-1^) as carbon source in culture media. Similarly, no statistically significant differences were found in X_m_ (X_glu_ = 10.99 ± 2.04 g/L; X_lac_ = 11.49 ± 0.76 g/L) (Fig 1a). However, it was observed that the morphology and diameter of the pellets formed in glucose (d = 2.3 ± 0.2 mm) added to the culture medium are 44% larger compared to the pellets obtained from the medium with lactose (d = 1.6 ± 0.2 mm; p < 0.05) (S1 Fig). Regarding the overall substrate consumption rate (q_s_), there is no significant difference between the use of glucose (q_s-glu_ = 0.26 ± 0.06 g sust/g biomass*d) or lactose (q_s-lac_ = 0.27 ± 0.03 g sust/g biomass*d) at a concentration of 150 mM total carbon (Fig 1b). In both glucose and lactose as carbon source in cultures, the initial pH was 6.5, and on the second day, a pH of 4–4.2 was reached. However, in the cultures where glucose was used, the pH remained within the mentioned range throughout the culture (Fig 1c). In contrast, in the cultures where lactose was used, there was a rebound to pH of 5.3 on day five and remained above 5.0 on day six (Fig 1c). From day 8 until the end of the culture, the pH stayed at levels similar to those seen in cultures where glucose was used as a carbon source.

**Table 1:** Stoichiometric and kinetic parameters of *C. coffeata* cultivation growth with glucose and lactose as carbon sources in shake flasks (30 °C, 150 rpm, 16 days).

Parameter	Glucose	Lactose
μ (d^-1^)	0.30 ± 0.00^a^	0.27 ± 0.03^a^
Maximum biomass (g/L)	10.99 ± 2.04^a^	11.49 ± 0.76^a^
Substrate consumption (g/L)	46.06 ± 5.24^a^	50.51 ± 5.81^a^
Yield (biomass/substrate) Y_x/s (gbiom/gsubstrate)_	0.24 ± 0.05^a^	0.23 ± 0.03^a^
Specific consumption rate q_s (gsubstrate/gbiomass day)_	0.26 ± 0.06^a^	0.27 ± 0.04^a^

p < 0.05, no significant difference

### Kinetic production of metabolites

**Fig 2:**
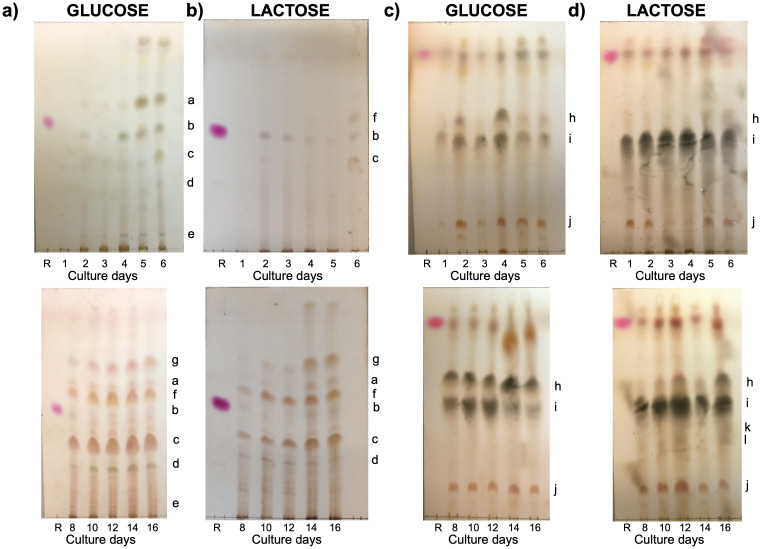
Comparison of the kinetic chromatographic profiles of chloroform extracts from *C. coffeata* biomass using glucose or lactose as the carbon source. Elution system CHCl_3_:MeOH (95:05), for compounds of low – intermediate polarity **(a and b)**; elution system CHCl_3_:MeOH (7:3) for more polar compounds **(c and d)**. R: Ursolic acid (reference terpene).

Since terpenoids are among the main secondary metabolites produced by basidiomycete fungi, kinetic monitoring of the profile of compounds extracted from the biomass was performed using an elution system designed for this type of molecule (Fig 2). Firstly, a change in the accumulation of various compounds produced by *C. coffeata* is observed as a function of time, both with glucose and lactose as carbon sources. Retention factors were determined in triplicate and average values with standard deviation were compared (S1 and S2 Table in S1 File). In the case of both glucose and lactose, it is observed that the components at 14 and 16 days do not show changes due to the appearance of other compounds of medium polarity.

When comparing the chromatographic profiles of the extracts obtained from biomass on different days using glucose or lactose, it was observed that after six days there is an accumulation of compounds in the glucose cultures (Component a, R_f_ = 0.65; d, R_f_ = 0.28; e, R_f_ = 0.07), which are not perceived, at the same time, in the culture with lactose. Likewise, a component (f, R_f_ = 0.58) accumulates earlier in cultures with lactose (6 days), although it appears later in cultures with glucose.

Once the cultures reached 14 days, the chromatographic profile in TLC in both carbon sources was very similar, when analyzing the intermediate polarity components with the CHCl_3_:MeOH (95:05) elution system. Taking into account that other common metabolites in fungi are alkaloids, it was decided to use a more polar system, CHCl_3_:MeOH (7:3) to compare the chromatographic profiles by TLC, finding that in cultures with lactose there are two differential components (k, R_f_ = 0.39 and l, R_f_ = 0.34) that are appreciated from day 8 of culture, which indicates that lactose also induces the synthesis of other compounds different from those presented in glucose.

**Fig 3:**
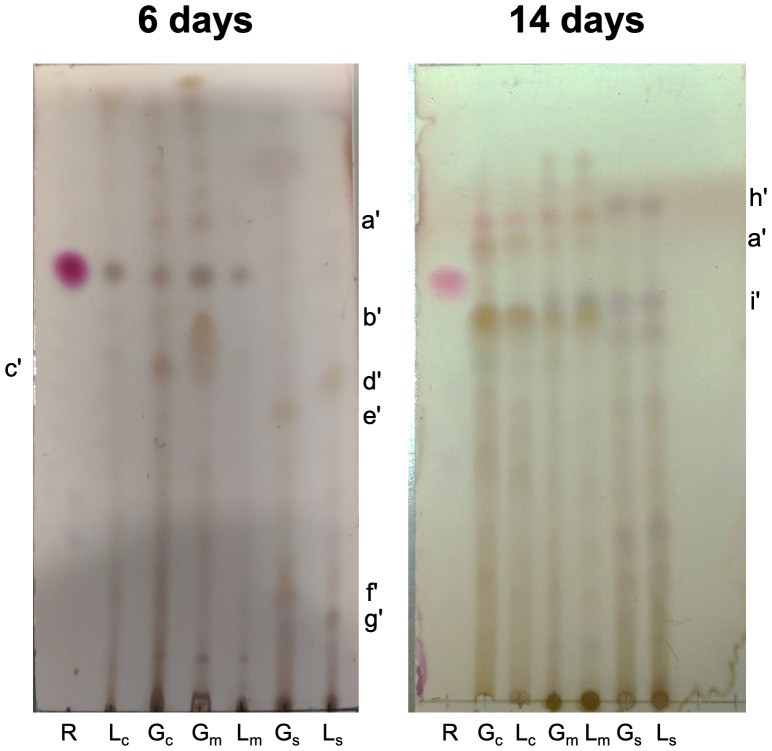
Chromatographic profiles of biomass extracts and supernatants from *C. coffeata* cultures. Elution system CHCl_3_:MeOH (95:05) x2. R: Ursolic acid (reference triterpene), G: glucose, L: lactose, subscripts c: chloroform extract, m: methanolic extract, s: chloroform extract from the supernatant.

After analyzing the above results and comparing them with the pH changes that occurred after 5 days in the lactose-containing cultures, it is noted that this coincides with the days when the differences in metabolite production with respect to glucose begin to be marked. Based on this, a comparative analysis was limited to 6 and 14 days of culture, examining both biomass and supernatant. The CHCl_3_:MeOH 95:05 elution system was used with double elution to improve resolution (see Fig 3, S3 Table in S1 File). In this analysis, it was observed that at 6 days, two components (d’, R_f_ = 0.51; g’, R_f_ = 0.14) were differentially detected in the culture supernatant using lactose. Similarly, in glucose, two different compounds are observed after 6 days (e’, R_f_ = 0.47; f’, R_f _= 0.20). However, later on, when the pH values were equal, no differences were observed between the supernatant profiles. To verify the presence of terpenoids in the extracts, TLC was conducted under the conditions shown in Fig 3, employing anisaldehyde as the developing agent (S2 Fig). Several components with a blue-violet hue, characteristic of terpenoids, were observed, confirming their presence in the extracts.

### Quantification of alkaloids and terpenoids

**Fig 4:**
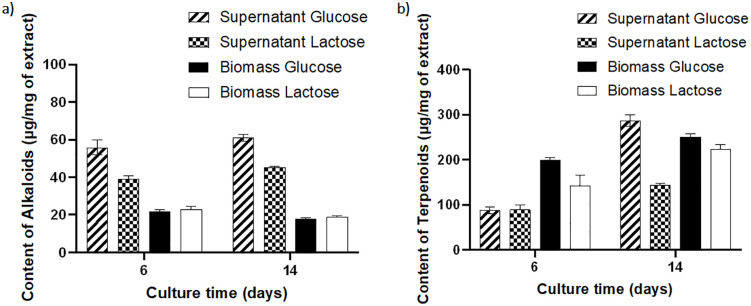
Quantification of secondary metabolites from *C. coffeata* cultures. Alkaloid contents **(a)** are reported as tryptophan equivalents; terpenoid contents **(b)** are reported as ursolic acid equivalents.

Based on these findings, we aimed to get an overview of alkaloid and terpenoid levels, so we quantified these compounds on days 6 and 14 using each carbon source. Alkaloids were determined as tryptophan equivalents, and it was observed that there was generally a greater accumulation of alkaloids in the culture supernatants compared to those obtained from biomass, for both carbon sources (Fig 4a). It was found that at 6 days of culture, the amount of intracellular alkaloids was similar between cultures supplemented with glucose and those with lactose. However, at 14 days, there was a slightly greater accumulation of intracellular alkaloids in the cultures where glucose was used, while the supernatants showed a greater amount of alkaloids in the cultures supplemented with glucose compared to lactose. On the other hand, a different finding was observed in the culture supernatants, as alkaloid accumulation was approximately one-third greater in cultures supplemented with glucose than in those supplemented with lactose at both 6 and 14 days of culture.

Terpenoids, on the other hand, were determined as ursolic acid equivalents (Fig 4b). In both carbon sources, a time-dependent accumulation of intracellular and supernatant terpenoids was observed. This, combined with the TLC results, appears to increase proportionally with biomass growth (Fig 2, Fig 4). In general, there was a higher amount of terpenoids in both the biomass and the supernatants of the glucose-treated cultures, at both 6 and 14 days of incubation.

### Fingerprint of extracts

To gain a broader understanding of the chemical composition of the extracts obtained from the fungal cultures, using each of the carbon sources, a fingerprint of the chloroform extracts obtained from the biomass was first obtained by obtaining ^1^H-NMR and APCI-MS spectra (S3 Fig). Resonance analysis revealed the presence of signals corresponding to methyls and methylenes (0.7–2.0 ppm), as well as olefinic protons (5.05 ppm), which corroborate the presence of terpenic compounds. It should be noted that this analysis did not demonstrate the presence of signals corresponding to alkaloids (10.0–11.0 ppm) in the chloroform extracts of biomass with either of the two carbon sources. Likewise, the 2.5 ppm signals correspond to DMSO, while the 3.3 ppm signal corresponds to residual water caused by DMSO hydration. In both culture conditions, it was found that most compounds have a mass/charge ratio (m/Z) between 300 and 600, as well as below 200 (m/Z), being very similar between them.

### Analysis by GC-MS

**Fig 5:**
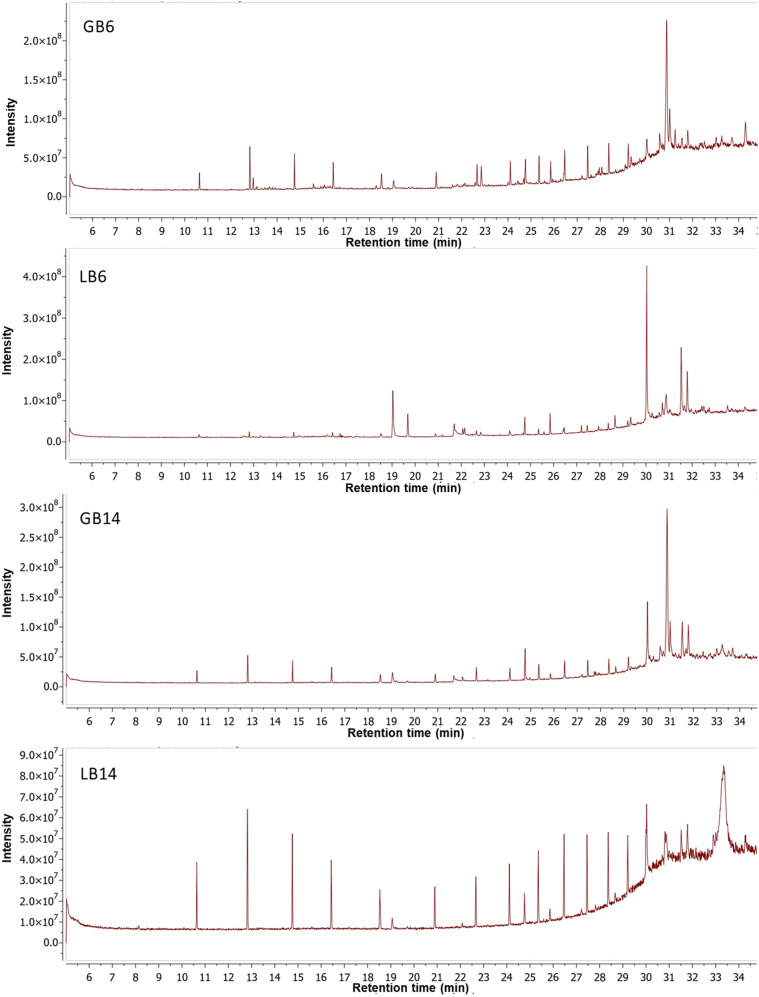
Chromatograms of chloroform extracts of *C. coffeata* biomass using glucose or lactose as the carbon source. GB: glucose biomass, LB: lactose biomass; the number refers to the number of days of cultivation.

**Fig 6:**
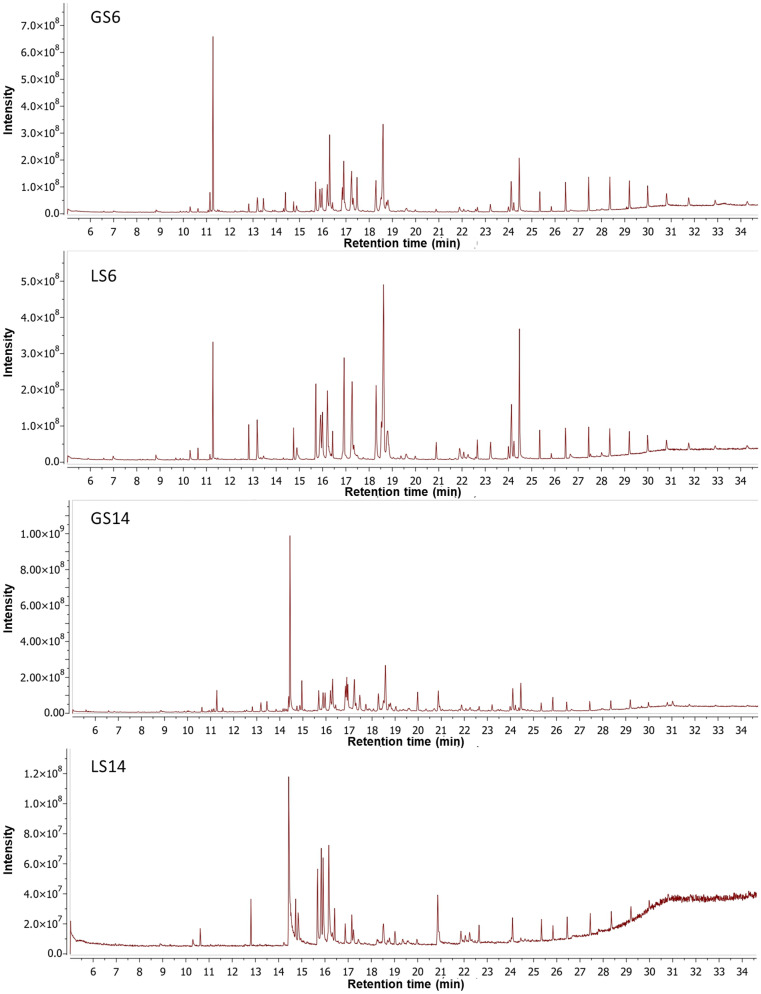
Chromatograms of chloroform extracts of *C. coffeata* supernatant using glucose or lactose as the carbon source. GS: glucose supernatant, LS: lactose supernatant; the number refers to the number of days of culture.

To identify some of the differential compounds produced by *C. coffeata* when using glucose or lactose as a carbon source in the culture medium, an analysis was performed using gas chromatography-mass spectrometry (GC-MS). Based on this analysis, it was possible to identify some compounds present in both the biomass (Fig 5) and the culture filtrate (Fig 6) of *C. coffeata* grown with both glucose and lactose in the culture medium. The results showed that, overall, a total of 77 compounds were detected: 43 in the biomass, 31 in the supernatant, and 3 compounds found in both biomass and supernatant (S4a Fig). Terpenoid-like compounds (based on their fragmentation pattern) and some other compounds were also detected in the biomass; in addition to possible terpenoids, alkaloids were also detected in the supernatants (S4b Fig).

A comparison parameter called “similarity index” between extracts was established, which consists of comparing, by means of a quotient, the compounds that are in common between the pair of extracts in question, taking into account that two identical extracts would have a value of 1 for this parameter (S5 Fig). Therefore, it was found that, regardless of the time and carbon source used in the medium, the biomass extracts were more similar to each other. Additionally, all supernatant extracts were more similar to each other than the biomass was to the supernatant, even under the same culture conditions and duration. This indicates a differential accumulation of intracellular compounds and those exported by *C. coffeata* to the supernatant. A notable finding in this analysis is that the extracts obtained after 6 days differ significantly from those corresponding to 14 days under the same carbon source, with values less than 0.3. This indicates a change of approximately 70% in the composition of the extracts due to the time factor.

**Fig 7:**
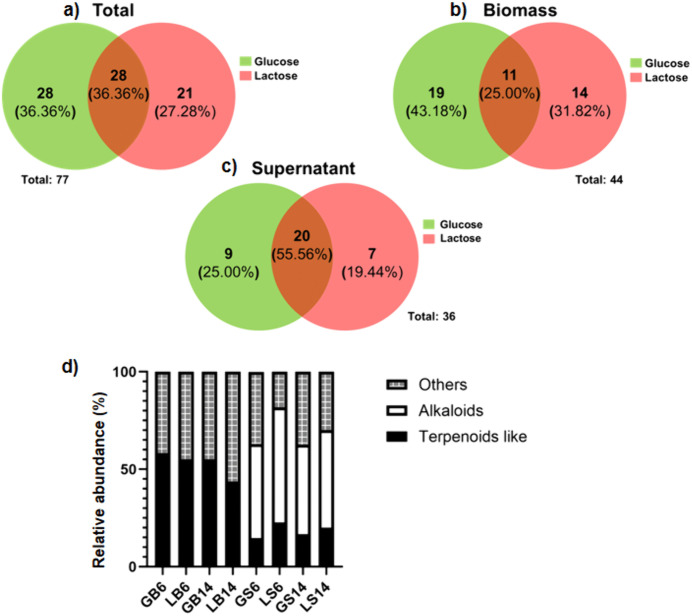
Comparison of the composition of chloroform extracts obtained from biomass and supernatant of *C. coffeata* cultures with glucose vs. lactose. Overall comparison of the components in the extracts **(a)**. Composition of the extracts of biomass **(b)**. Composition of the extracts of supernatant **(c)**. Relative abundance of secondary metabolites in each of the extracts **(d)**. G: glucose; L: lactose; B: biomass; S: supernatant; 6: six days of culture; 14: fourteen days of culture.

When analyzing the differences between compounds produced by *C. coffeata* using glucose or lactose as a carbon source, it was found that 36.36% of the compounds (28 compounds) are similar under both conditions. The remaining compounds are different, with 23 compounds (27.27%) observed when cultivated with lactose and 28 compounds (36.36%) when cultivated with glucose (Fig 7a). Chromatographic analysis (Fig 6) of the compounds extracted from the *C. coffeata* biomass revealed 11 compounds in common with both carbon sources, including ergosterol (T_R_: 30.87 min); ergosta-7,22-dien-3-ol (T_R_: 31.04 min); and an ergosterol derivative (T_R_: 31.79 min). Regarding the compounds observed only in the biomass of the glucose culture (19 compounds), linoelaidic acid (T_R_: 21.69 min, 14 days of culture) is found in first place. This fatty acid is commonly found in the seeds of some plants but is little reported in fungi. It was also possible to observe an ergosterol-derived compound (T_R_: 30.57), which was only observed after 14 days in the glucose culture.

Other compounds were also present that could not be compared with any database. However, they exhibited a fragmentation pattern in mass spectrometry similar to that of terpenoids, suggesting a potential relationship to this group of molecules. These compounds were therefore assigned as a group called terpenoid-like compounds. For example, a compound that presented a T_R_: 32.73 min, which presents a fragmentation pattern characteristic of triterpenoids. In the case of biomass obtained from lactose, 14 differentially produced compounds were identified. In this case, none of the differential molecules were identified. However, 12 of them exhibit a fragmentation pattern consistent with terpenoid-type compounds.

The analysis of supernatants from *C. coffeata* cultures with two carbon sources at different times showed production of alkaloids, not seen in the biomass. The alkaloid compounds present with both carbon sources were 3-methyl-6-(1-methylethyl)-2,5-piperazinedione (T_R_: 14.89 min), N-acetyl-3-methyl-1,4-diazabicyclo[4.3.0]nonan-2,5-dione (T_R_: 15.70 min), (3S,6S)-3-butyl-6-methylpiperazine-2,5-dione (T_R_: 15.97 min), hexahydro-pyrrolo[1,2-a]pyrazine-1,4-dione (T_R_: 16.20 min), 3,6-Diisopropylpiperazine-2,5-dione (T_R_: 17.24 min), hexahydro-3-(2-methylpropyl)-pyrrolo[1,2-a]pyrazine-1,4-dione (T_R_: 18.29 min), 3,6-bis(2-methylpropyl)-2,5-piperazindione (T_R_: 18.52 min), hexahydro-3-(2-methylpropyl)-pyrrolo[1,2-a]pyrazin-1,4-dione (T_R_: 18.59 min), 5,10-Diethoxy-2,3,7,8-tetrahydro-1H,6H-dipyrrolo[1,2-a:1’,2’-d]pyrazine (T_R_: 18.80 min). The compounds that were differentially detected in the extracts of the supernatants when cultivated with glucose were 1H-Indole-3-carboxaldehyde (T_R_: 16.84 min), which appeared only at 6 days but was no longer observed at 14 days, and a terpenoid-type compound (T_R_: 19.98 min). On the other hand, in the extracts of the supernatants of the cultures with lactose, the compounds cis-3a,4,7,7a-tetrahydro-1H-Isoindole-1,3 (2H) -dione (T_R_: 13.19 min) were differentially obtained, in addition to two terpenoid-type compounds (T_R_: 22.24 min and T_R_: 24.24 min). Aside from the terpenoid and alkaloid compounds, 1-octacosanol was produced (TR: 30.02 min). It appears early in *C. coffeata* biomass when using glucose, but when lactose is used, it appears near the end of cultivation.

## Discussion

As expected, a differential production of compounds, as well as a distinct accumulation of alkaloids and terpenoids, was observed in *C. coffeata* when using glucose or lactose as a carbon source in the culture medium, as evidenced by TLC and GC-MS (Figs 1, 5, and 6). The accumulation of different terpenoids depending on the carbon source used has been reported in *Ganoderma lucidum*, which is the most important basidiomycete in the field of secondary metabolite production [11,12]. However, the explanation of the relationship between the carbon source and the regulation of secondary metabolite production is unclear. Although it has been suggested that global regulation by the carbon source plays essential roles, as reported for CreA/CRE1 in *Aspergillus flavus*, *A. nidulans,* and *Trichoderma reesei*, among others [25–27]. In *G. lucidum*, a functional homolog of CreA (termed GlCreA) has been identified [28]. The appearance of different compounds in both glucose and lactose as carbon source in the case of *C. coffeata* suggests that regulation occurs through more complex regulatory mechanisms [29]. For this reason, monitoring observable changes in culture is crucial to gaining a better understanding of the behavior of secondary metabolite production by basidiomycetes.

No significant statistical differences were observed in the growth of *C. coffeata* when cultured with glucose or lactose as the carbon source. However, it was noted that the production of compounds varied over time, as evidenced initially by TLC (Fig 2). Likewise, differences in the pellet sizes were observed in submerged cultures using glucose versus lactose (S1 Fig). Pellet morphology has been suggested to play an important role in the production of specific secondary metabolites [22,30]. For example, the production of citric acid (in *Aspergillus niger*) and fumaric acid (*Rhizopus oryzae*) depends on the pellet morphology, as a consequence of the cultivation time [30,31]. Zhang et al. [22] evaluated the changes in the morphology of *Monascus purpureus* pellets using various carbon sources. They found that the use of citric acid resulted in 50.52% larger pellets and 58.51% greater biomass compared to glucose. However, the pellets obtained with citric acid showed signs of cell lysis in their internal structure, which may influence the production of secondary metabolites. In that same study, the production of the polyketide citrinin was evaluated in pellets with different diameters, demonstrating that there is a direct correlation between the pellet diameter and the citrinin production, where the pellets with a diameter of 2.04 mm showed a production of approximately three times the reference condition (pellet of 1.7 mm), while when reducing the pellet size to 1.4 mm, the production decreased by approximately half compared to the reference condition [23]. The above suggests an important relationship between pellet formation and size, as well as the amount of secondary metabolites produced, and their relationship with the carbon sources used. One possible explanation for this phenomenon may be related to the mass transfer of nutrients to the mycelium found in the innermost layers of the pellet, which have a different metabolic response than the inner layers due to lower nutrient availability [32,33].

Another observation was an increase in pH between days 4 and 6 (Fig 1c) in cultures with lactose, compared to those with glucose. This is notable because pH increases observed in microbial/fungal cultures have been linked to the release of nitrogenous compounds derived from amino acid metabolism into the supernatant, which in turn correlates with enhanced production of nitrogen-rich secondary metabolites. For instance, in *Aspergillus spp.* The extracellular pH plays a crucial role in the production of secondary metabolites, such as aflatoxin and sterigmatocystin [34].

This work focused on quantifying alkaloids and terpenoids, the most common fungal secondary metabolites relevant for potential applications [2,17,35]. the accumulation of alkaloids mainly occurred in the culture filtrate for both carbon sources (Table 1), as expected, since alkaloids are highly polar and are often exported into the supernatant via extracellular vesicles in fungi [36]. Terpenoids are the most abundant secondary metabolites produced by fungi; therefore, both quantification and TLC monitoring focused on them, indicating that accumulation was greater in cultures using glucose as the carbon source compared to those using lactose. While the TLC component analysis focused primarily on the search for terpenoid-like molecules, changes in other compounds cannot be ruled out, as observed in kinetic production monitoring. It has been reported that the carbon and nitrogen sources play significant roles in the production and diversity of secondary metabolites [14,15,17]. This is related to the accumulation of precursors that feed the different metabolic pathways. The synthetic pathways of alkaloids are nourished by various precursors, among which the amino acids (true alkaloids) stand out, which are the main base of these molecules, and the non-amino acid precursors. (pseudoalkaloids) [6,37]. Among the main precursor amino acids are lysine, tryptophan, phenylalanine, and tyrosine, but there are also alkaloids produced from alanine, aspartate, ornithine, and histidine, so the accumulation of these molecules in particular above typical concentrations is key in the synthesis of true alkaloids [6]. Conversely, pseudoalkaloids are produced through transamination to a receptor structure, resulting in the final molecule. The primary donor molecules are derivatives of phenylalanine, purines, and pyrimidines [37]. In the case of macroscopic fungi, it has been reported that the most common fungal alkaloids are those derived from indoles and isoxazoles, however other types of alkaloids have been described in basidiomycetes such as pyrrole derivatives, pyrazine derivatives, and those of mixed biosynthetic origin [5,6,37–39]. For example, a unique family of alkaloids, called lucidimines, has been identified in *Ganoderma lucidum*, of which four compounds have currently been described: Lucidimines A – D [38]. Likewise, iridoid alkaloids possess a monoterpenoid fragment (iridoid motif) and an alkaloid region derived primarily from amino acids or transaminations. In the case of lucitamines, a monoterpenoid region is observed in the structure (similar to an iridiod), but their biogenesis, especially regarding the alkaloid region, is not clear. However, their dependence on the accumulation of the monoterpenoid fraction is evident [6,40]. Another example is the case of purupurolic acid, which originates from the transamination of pyruvate to form alanine, its direct precursor, in the fungus *Claviceps purpurea*. It should be noted in this case that a greater accumulation of pyruvate results in a greater amount of alanine, which in turn is directed towards the synthesis of purupurolic acid [5].

Terpenoids all share a common precursor, which in turn originates from pyruvate: isopentenyl pyrophosphate (IPP) and dimethylallylpyrophosphate (DMAPP), which polymerize to lengthen the chain and give rise to the various terpenoids. Therefore, the accumulation of pyruvate is crucial in the synthesis of terpenoids, and the diversification of molecules of this type derives from the activation and inactivation of the different terpene synthase genes [17]. A clear link exists between the buildup of key metabolic precursors and the production of secondary metabolites. For this reason, monitoring metabolite production at the global level is of interest in the search for secondary metabolites using the OSMAC strategy [15]. Extract fingerprinting enables a rapid comparison of the primary metabolites in the microbial culture. In our case, APCI-MS was chosen because it is a technique that allows gentle ionization of the molecules present in the sample for subsequent analysis in the mass spectrometer. Since the adduct formed in atmospheric pressure chemical ionization typically has a single charge [M-H^+^], it can be inferred that each signal in the analyzed extracts corresponds to a different compound [41]. In general, the extracts are very similar in terms of the signals present, indicating a high similarity between them. For this reason, it was decided to perform a more detailed analysis, using GC-MS, to obtain more information on the compounds produced under the different culture conditions. It was observed that some compounds were similar between the extracts evaluated, which led to proposing a comparison measure of the similarity of the extracts (similarity index) calculated by comparing the same compounds present in two different extracts and using a quotient dividing them by the total (S5 Fig). It was observed that the most similar extracts were those from the supernatants, where alkaloids and terpenoid-like compounds were found. This indicates that the compounds exported by the fungus are predominantly the same. However, they still contain some distinct compounds that may be relevant when searching for new molecules, either because they exhibit biological activity or have synergistic effects with other active molecules.

Conversely, the differences among biomass extracts are more pronounced, indicating variations in secondary metabolism activity, which could be linked to precursor accumulation, as previously noted. Since, as observed at 6 days, a series of compounds begin to accumulate, as evidenced by TLC (Fig 2), they possibly give rise to those detected 14 days later. In other words, the compounds present at 6 days may act as precursors to other molecules. It can further be inferred that during culture, a response to decreases in nutrient concentration and the accumulation of metabolic by-products alters the profile of the compounds produced. This response may account for the observed differences [42,43].

Among the most relevant molecules identified are those of a different origin than alkaloids and terpenoids, such as linoelaidic acid, a fatty acid found in the seeds of some plants but rarely reported in fungi. This compound has been reported to be relevant in mammalian nutrition for forming prostaglandins and components of the cell membrane, as well as participating in cell signaling [44] and exhibiting anticancer potential [45]. Likewise, 1-octacosanol is a molecule of interest for production, as it has been reported to exhibit anti-inflammatory, antibacterial, antifungal, and antioxidant effects [46]. This highlights the importance of selecting the appropriate carbon source in the culture medium to direct production toward the desired metabolites.

The appearance of alkaloids of indole origin was expected because they were common in fungi. However, alkaloids derived from piperazine have been found, which are not commonly found in nature but have been reported since the 1970s in some fungi, such as *Penicillium herquei* [47]. The synthesis of these types of structures is generally uncommon, and both piperazine and its derivatives are usually obtained artificially through organic synthesis. Therefore, the identification of these molecules produced by *C. coffeata* is of great interest. Since the main biological activity reported for piperazines is as anthelmintics, these molecules may be relevant in this application or even act as scaffolds for modifications or synthesis precursors [9,10].

Furthermore, 13 terpenoid-like molecules were differentially expressed when glucose was used as the carbon source, while 10 (8 in biomass and 2 in supernatants) were observed when lactose was used. This reinforces the idea that a key factor is the accumulation of precursors, surely pyruvate, to feed the various secondary metabolite pathways when glucose serves as the carbon source [48]. However, with lactose, it was possible to detect other compounds that are differential but less abundant. Using different carbon sources affects the regulation of secondary metabolite production and is important to consider when searching for new molecules. [17]. Since in the compounds that are produced differentially, the regulation may be due to the level of gene transcription, since as has been seen in other fungal models, such as *Fusarium*, the synthesis of trichothecenes (sesquiterpenoids) responds differentially to the recognition of the structure of the carbohydrates used in the culture medium supplement and to the presence (or absence) of a particular monomer [49,50]. This is because it is not only a matter of providing similar amounts of carbon sources for secondary metabolite production. Their interaction with and recognition by cells can trigger specific signaling that leads to the production of different metabolites. Similarly, the factor of metabolite production as a function of time is important. When searching for molecules with applications, primarily in the pharmacological field, these molecules can exhibit different effects depending on their structure, the presence of functional groups, and other modifications they may undergo during culture time.

These results suggest that the differential production of alkaloids and terpenoids is due to precursor accumulation, rather than global regulation by CRE1, resulting from glucose utilization. Most compounds are present with both carbon sources, but overall concentrations vary. In conclusion, the production of alkaloids and terpenoid-like molecules, among other secondary metabolites, in *C. coffeata* exhibits differences in the accumulation of these compounds, as well as differential production of some compounds. Furthermore, the kinetic production of compounds is relevant for obtaining specific molecules, as many of these molecules may be precursors of others and thus their trace is lost throughout the culture, as observed in the similarity comparisons of extracts with the same carbon source. Additionally, the pellet size may have a primary relationship with the production of secondary metabolites. Thus, selecting the optimal time for cultivation, as well as the suitable carbon source, is a crucial factor in the search for new molecules with potential applications as drugs or synthetic precursors.

## Supporting information

S1 Fig Comparison of *C. coffeata* growth in flasks. 30 °C, 150 rpm, 14 d. a) Growth morphology of *H. coffeata* on glucose (left) or lactose (right). b) Cumulative pellet sizes obtained with glucose (white) or lactose (black).
(TIF)

S2 Fig Chloroform extracts of the biomass and supernatant revealed with anisaldehyde. Elution system CHCl_3_:MeOH (95:05). R: Ursolic acid (reference triterpene), G: glucose, L: lactose, subscripts c: chloroform extract, m: methanolic extract, s: supernatant extract.
(TIF)

S3 Fig Fingerprint of chloroform extracts of *C. coffeata* biomass at 14 days of culture. Left: glucose, right: lactose; top: 1H-NMR spectrum; bottom: mass spectrum obtained using APCI-Mass.
(TIF)

S4 Fig Relative abundance of all secondary metabolites detected in the biomass and supernatant extracts. (TIF)

S5 Fig Similarity index of components between the various extracts. (TIF)

S1 File Dataset used in analysis. (XLSX)
